# Somatic gonad morphogenesis in *C. elegans* requires the heterochronic pathway acting through HBL-1

**DOI:** 10.1093/g3journal/jkaf170

**Published:** 2025-07-28

**Authors:** Madeleine Minutillo, Kevin Kemper, Maria Ivanova, Erika Pianin, Eric G Moss

**Affiliations:** Department of Molecular Biology, Graduate School of Biomedical Sciences, Rowan University, Stratford, NJ 08084, United States; Department of Molecular Biology, Graduate School of Biomedical Sciences, Rowan University, Stratford, NJ 08084, United States; Department of Molecular Biology, Graduate School of Biomedical Sciences, Rowan University, Stratford, NJ 08084, United States; Department of Molecular Biology, Graduate School of Biomedical Sciences, Rowan University, Stratford, NJ 08084, United States; Department of Molecular Biology, Graduate School of Biomedical Sciences, Rowan University, Stratford, NJ 08084, United States

**Keywords:** *Caenorhabditis elegans*, gonad morphogenesis, heterochronic genes, nematode development, RNA-binding, zinc fingers, WormBase

## Abstract

Heterochronic genes are known for acting in succession to control the timing of stage-specific events of the developing *Caenorhabditis elegans* larva. While most heterochronic mutants have normal gonad development, in a few cases, defective timing regulators lead to variations in gonad development, although with little to no effect on fertility. We found that a double mutant of 2 heterochronic genes, a *lin-28* null allele and *hbl-1* hypomorphic allele, results in a catastrophic failure of gonad morphogenesis resulting in sterility. This defect includes a high-penetrance disruption of normal gonad arm migration as well as frequent absence of 1 or both spermathecae. We demonstrate that the abnormal gonad morphology and novel sterility phenotype is ultimately due to loss of *hbl-1* activity in larval development. To clarify the mechanism of how *lin-28* positively influences *hbl-1* activity, we demonstrate a direct interaction between the RNA-binding protein LIN-28 and the 5′UTR of *lin-46*, and in turn, a direct interaction between LIN-46 and 2 zinc fingers of HBL-1. Nevertheless, our genetic analysis indicates that *lin-46* accounts for only part of *lin-28*'s regulation of *hbl-1* and that some portion of *lin-28*'s effect is independent of *lin-46*.

## Introduction

Heterochronic genes of the nematode *Caenorhabditis elegans* comprise a well-characterized regulatory system explicitly controlling timing during tissue and organ formation in animals (Moss 2007; Rougvie and Moss 2013). This genetic pathway—which is comprised of about a dozen genes including several that encode microRNAs—governs the succession of cell division and differentiation events in several tissues during *C. elegans* larval development. Most tissues in this animal that develop post-embryonically execute specific developmental events at each larval stage. Mutations in heterochronic genes cause stage-specific events to be either skipped or repeated, resulting in precocious or reiterative phenotypes, respectively. For example, loss of either *lin-28* or *hbl-1* activities causes animals to skip L2 developmental events and to precariously execute events of the L3 and later stages (Moss et al. 1997; Abrahante et al. 2003; Abbott et al. 2005). Loss of *lin-46* activity, however, causes reiteration of L2 events (Pepper et al. 2004). The most significant consequence of such abnormal timing for the worm is an asynchrony between the development of the egg laying system, which emerges from the hypodermis and other tissues, and the gonad development, which occurs on its normal schedule in these mutants.

The majority of heterochronic mutants are fertile with properly formed and fully functional gonads. Certain mutations cause notable defects in the path of migration of the distal tip cells (DTCs) as the gonad grows and takes shape (Tennessen et al. 2006; Cecchetelli and Cram 2017). For example, particular alleles of *daf-12* cause both reiterative hypodermal development as well as an abnormal gonad shape where the DTCs take aberrant paths during gonad growth (Antebi et al. 1998; Hammell et al. 2009). The DTCs normally turn dorsally at the third larval stage (L3) molt but often fail to do so in these mutants. Despite the deviant migration, *daf-12* mutant animals have undisturbed fertility. *daf-12* encodes a nuclear hormone receptor that regulates the transcription of microRNA genes involved in heterochronic gene regulation (Hammell et al. 2009).

Choi and Ambros (2019) documented LIN-28's role in the normal morphogenesis of the hermaphrodite spermatheca, the part of the somatic gonad where sperm are held and fertilization takes place. Mutations in genes causing structural abnormalities that affect ovulation, fertilization, spermathecal exit, and egg laying reduce the overall fertility of an animal (Kovacevic and Cram 2010; Choi and Ambros 2019). Choi and Ambros (2019) also observed that RNAi of *hbl-1* caused a similar spermathecal defect. However, it is not clear how *lin-28* or *hbl-1* affects spermathecal development or whether that effect is related to their roles in the developmental timing pathway.

Mutations in either *lin-28* or *hbl-1* cause widespread precocious hypodermal development, where events of the second larval stage (L2) are skipped (Ambros and Horvitz 1984; Abrahante et al. 2003; Lin et al. 2003). LIN-28 is a conserved RNA-binding protein that controls events of the L2 stage via post-transcriptional regulation of *hbl-1* at least in part via the negative regulation of *lin-46* (Pepper et al. 2004; Vadla et al. 2012; Ilbay and Ambros 2019). A second activity of LIN-28 blocks the maturation of the pre-let-7 microRNA by which it controls events of the L3 stage (Vadla et al. 2012). HBL-1 is a zinc finger transcription factor belonging to the Ikaros family (Fay et al. 1999). Null alleles of *hbl-1* die as late-stage embryos or young larvae because *hbl-1* is required for hypodermal differentiation late in embryogenesis (Fay et al. 1999; Abrahante et al. 2003; Lin et al. 2003). Hypomorphic alleles of *hbl-1* have been found that survive to adulthood but show precocious heterochronic defects, specifically skipping events of the L2, much like *lin-28* null alleles (Abrahante et al. 2003; Lin et al. 2003). *hbl-1* is negatively regulated by let-7 family microRNAs acting through its 3′UTR (Abbott et al. 2005).

Numerous genetic studies have placed *hbl-1* downstream of *lin-28*, and it is likely the most direct regulator of L2 fates of the known heterochronic genes. Because null alleles of *hbl-1* die as embryos, studying its postembryonic role has been a challenge that has relied on hypomorphic alleles, therefore, some genetic tests that typically rely on null alleles have been difficult to evaluate. *hbl-1* activity in the heterochronic pathway appears to be regulated both posttranscriptionally and posttranslationally. Through its 3′UTR, *hbl-1* expression is regulated by members of the let-7 family of microRNAs, where deletion of either the microRNAs or the 3′UTR causes misexpression of the protein. Furthermore, LIN-46 appears to affect HBL-1 localization (Vadla et al. 2012; Ilbay and Ambros 2019). Here, we provide evidence for a direct association between LIN-28 and the *lin-46* mRNA as well as between LIN-46 and HBL-1 that appears to alter HBL-1's activity.

Because *lin-28* and *hbl-1* both control L2 events, we attempted to sort out whether *lin-28* and *hbl-1* act in a linear pathway to control hypodermal development or whether the pathway branches at some point. We made an unexpected observation concerning gonad morphogenesis that nevertheless helps us answer that question.

## Materials and methods

### Culture conditions

Nematodes were grown under standard conditions at 20 °C unless otherwise indicated (Sulston and Horvitz 1977). Strains with multiple allelic mutations were generated by following lesions via PCR and confirmed through DNA sequencing. Most strains contain the wIs78 transgene, a derivative of wIs1, which contains seam cell nuclei (*scm::GFP*) and seam cell junction (*ajm-1::GFP*) markers to identify and quantify lateral hypodermal seam cells (Koh and Rothman 2001). For gonad analysis, males were produced by growing strains on *Escherichia coli* bacteria containing the RNAi plasmid pLT651 (Timmons et al. 2014).

### Strains used

N2 wild type

RG733  *wIs78 (ajm-1::GFP & scm::GFP)*
ME414 *wIs78; hbl-1(ae23) X*
ME419 *wIs78; lin-28(ga54) I*
ME433 *wIs78; lin-28(ga54) I; lin-46(ma174) V; hbl-1(ae23) X*
ME438 *wIs78; lin-46(ae30) V*
ME443 *wIs78; lin-28(ga54) I; hbl-1(ae23)/tmC30[myo-2p::Venus]X*
ME452 *wIs78; lin-28(ga54) I; hbl-1(ve18)/tmC30[myo-2p::Venus]X*
ME455 *wIs78; lin-28(ga54) I; qIs56[lag-2p::GFP + unc-119(+)] V; hbl-1(ae23)/*
*tmC30[myo-2p::Venus]X*
ME456 *wIs78; lin-28(ga54) I; ezIs2[fkh-6::GFP + unc-119(+)] III; hbl-1(ae23)/*
*tmC30[myo-2p::Venus]X*
ME481 *wIs78; lin-28(ga54) I; qIs56[lag-2p::GFP + unc-119(+)] V*
ME485 *wIs78; lin-28(ga54) I; ezIs2[fkh-6::GFP + unc-119(+)] III*
ME502 *cshIs140 [rps-28pro::TIR-1(F79G)_P2A mCherry-His-11;*
*Cbunc-119(+)] II; HBL-1-AID (hbl-1 (aeIS8 (12.4), HBL-1::AID)); wIs78 (pDP#MM016B (unc-119) + pJS191 (AJM-1::GFP + pMF1 (SCM::GFP) + F58E10)*
ME509 *wIs78; lin-28(ga54) I; arTi145 [ckb-3p::mCherry::his-58::unc-54 3′UTR] II; hbl-1(ve18)/tmC30[myo-2p::Venus]X*
ME521 *wIs78; lin-28(ga54) I; cshIs140 [rps-28pro::TIR-1(F79G)_P2A mCherry- His-11; Cbunc-119(+)] II; HBL-1-AID (hbl-1 (aeIS8 (12.4), HBL-1::AID))*
ME522 *wIs78; lin-28(ga54) I; lin-46(ma174) V; cshIs140 [rps-28pro::TIR-1(F79G)_P2A mCherry-His-11; Cbunc-119(+)] II; HBL-1-AID (hbl-1 (aeIs8 (12.4), HBL-1::AID))*
ME524 *hbl-1(ve18)X; ezIs2[fkh-6::GFP + unc-119(+)] III*
ME525 *hbl-1(ve18) X; qIs56[lag-2p::GFP + unc-119(+)] V*
ME542 *wIs78; lin-46(ae30) V; hbl-1(ae23) X*
ME543 *wIs78; lin-46(ae30) V; hbl-1(ve18) X*
ME551 *wIs78; lin-28(ga54) I; lin-46(ma174) V; hbl-1(ve18) X*
ME568 *unc-55(e1170); nuIs279 aeEx49 (Punc-25:lin-46, ttx-3::GFP)*
ME569 *unc-55(e1170); nuIs279 aeEx50 (Punc-25:moc-1, ttx-3::GFP)*
KP5348  *nuIs279 [Punc-25:unc-57:GFP + Punc-25:mCherry:rab-3]*
KP5363 *unc-55(e1170); nuIs279*
KP6119 *unc-55(e1170); hbl-1(mg285); nuIs279*

### Microscopy

Nomarski DIC and fluorescence microscopy were used for quantitative and qualitative assessments of phenotypes. Developmental stage was assessed by time from hatching, extent of gonad development, and, in some cases, counting molts. Images were acquired with AxioCam with AxioVision with Plan-NEOFLUAR 63× and alpha Plan-FLUAR 100× objectives on a Zeiss Axioplan2 microscope.

### RNA interference

Bacterial-mediated RNA interference was performed as previously described (Timmons et al. 2014). The RNAi vectors used contain a 3.5 kb region of *hbl-1* genomic sequence in the T444T backbone (Sturm et al. 2018). Bacteria were induced in culture and seeded on nematode growth media (NGM) plates containing 1 mM ITPG (GoldBio) and 50 μg/ml ampicillin (VWR Life Science) and 12.5 μg/ml tetracycline (FisherBiotech).

### Auxin-inducible degron

The auxin-inducible degron (AID)-tagged *hbl-1* allele was constructed using the CRISPR/Cas9 as described using the pDD162 vector and *dpy-5* as a co-CRISPR marker (Dickinson and Goldstein 2016; El Mouridi et al. 2017 ). The gRNA was synthesized using Invitrogen’s MEGAshortscript T7 Transcription Kit and the repair template was generated by the following primers:

5′ ATCAGGCTTTCAATGAGCTAAGCTTTGCTCTCCACATGTAtCAgGCgcGtCAtC Agatgcctaaagatccagccaaacc
5′ TTAACGAGGACGTCCTCATTATTGGTGTCTGGCTTGGTACTTActtcacgaacgccg ccgc

Repair templates were mixed and heated to 96 °C for 5 min and then placed on ice (Dokshin et al. 2018). The sequence targeted the 3′ end of the *hbl-1* protein coding region. The guide RNA sequence used targeted the top strand: 5′ CATGTACCAAGCCAGACACC. 5-Ph-IAA was resuspended in DMSO to make 10 mM stocks (Hills-Muckey et al. 2021). Animals were grown on 2 variations of NGM plates: plates treated with 1 μM 5-Ph-IAA (Sigma-Aldrich), or plates treated with 1 μM 5-Ph-IAA, 1 mM ITPG (GoldBio), 50 μg/ml ampicillin (VWR Life Science), and 12.5 μg/ml tetracycline (FisherBiotech).

### Yeast 2- and 3-hybrid assays

Yeast 2-hybrid assays were performed as described previously (Golemis et al. 2008). The *lin-46* open reading frame was fused to the DNA-binding domain in pMW103. Portions of the *hbl-1* open reading frame were fused to the activation domain in pJG4-5. These plasmids were co-transformed with the *lacZ* reporter. X-gal overlays were assessed after 6 h and overnight.

Yeast 3-hybrid assays were performed using the YBZ-1 strain as described previously (Hook et al. 2005). The *lin-28* open reading frame was fused to the activation domain sequence in pACT2, and experimental RNAs were fused to the MS2 stem loop sequence in pIIIA/MS2-2. X-gal overlays were assessed after 6 h and overnight.

### Statistics

Statistical *P-*values presented in the text and in Supplementary Table 2 were calculated as follows: % fertile (Fisher's exact test). average brood size (unpaired parametric *t*-test), and number of seam cells (nonparametric Mann–Whitney test). For each outcome type (19 pairwise tests), a Bonferroni correction was used to set the significance level: *P* < 0.05/19 = 0.0026.

## Results

### Fertility requires the combined activities of *lin-28* and *hbl-1*

**Fig. 1.**
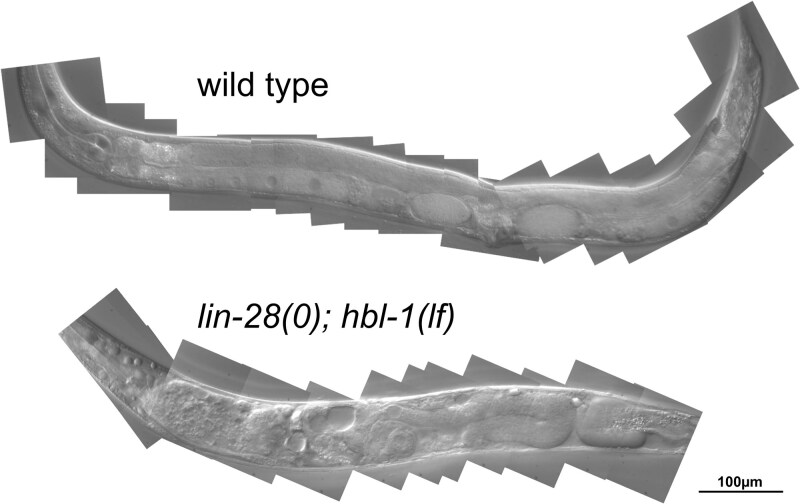
Gross terminal phenotype of *lin-28; hbl-1* double mutant of *C. elegans.* Top: wild-type *C. elegans* adult with mature oocytes and 2 eggs in the uterus. Anterior to the left and dorsal up. Bottom: a similarly aged *lin-28(0); hbl-1(lf)* animal, displaying an overall “dumpy” morphology, apparent lack of vulva, disorganized gonad, vacuoles, yolk deposits, and sterility.

The genes *lin-28* and *hbl-1* are required for normal L2 development, with mutations in each causing animals to skip L2-specific events in the hypodermis (Moss et al. 1997; Abrahante et al. 2003; Abbott et al. 2005). As part of addressing the relationship between these 2 regulators, we attempted to construct a double mutant between a null allele of *lin-28*(*ga54*) and a hypomorphic allele of *hbl-1(ve18),* which individually show only a heterochronic phenotype (Abrahante et al. 2003). We found that animals homozygous for both alleles were sterile and lacked the characteristic protruding vulva of each alone (Fig. 1). Occasionally, some double mutants were able to produce offspring, but the fertility and fecundity of these animals was severely reduced relative to the single mutants alone (Table 1, columns A and B, line 5 vs lines 2 and 3: *P* < 0.0001). However, we could maintain a balanced strain containing both alleles when *lin-28(ga54)* was homozygous and *hbl-1(ve18)* was heterozygous; all double mutants we characterized were offspring of such balanced strains.

**Table 1.** Phenotypes of mutant strains.

		A	B	C
	Genotype^a^	% fertile^b^	Average brood size of fertile animals (*n*)^b,c,d^	Number of seam cells at adulthood (*n*)^b,c^
1	Wild type	100	262.7 (10)^+^	16.1 (30)
2	*lin-28(ga54)*	100	20.3 (57)	10.8 (28)
3	*hbl-1(ve18)*	95	48.4 (44)	20.9 (27)*^e^*
4	*hbl-1(ae23)*	100	248.9 (24)^+^	16.1 (31)
5	*lin-28(ga54); hbl-1(ve18)*	10.6	3.4 (66)	10.4 (21)
6	*lin-28(ga54); hbl-1(ae23)*	19	1.92 (68)	11.1(22)
7	*lin-28(ga54); lin-46(ma174); hbl-1(ve18)*	65	4.5 (40)	12.6 (46)
8	*lin-28(ga54); lin-46(ma174); hbl-1(ae23)*	92	10.7 (62)	15.8 (37)
9	*lin-46(ae30)*	100	56.5 (36)	12.1 (31)
10	*lin-46(ae30); hbl-1(ve18)*	82.8	9.6 (35)	13.5 (35)
11	*lin-46(ae30); hbl-1(ae23)*	100	48.4 (35)	12.2 (34)
12	*hbl-1(AID)*, no auxin	100	323.5 (10)^+^	16.1 (36)
13	*hbl-1(AID)*	100	51.1 (15)	14.1 (36)
14	*hbl-1(AID + RNAi)*, no auxin	91	12.1 (43)	12.2 (24)
15	*hbl-1(AID + RNAi)*	50	6.9 (36)	11.8 (26)
16	*lin-28(ga54); hbl-1(AID + RNAi)*	0	0 (39)	10.9 (39)
17	*lin-28(ga54); lin-46(ma174); hbl-1(AID + RNAi)*	27	2.9 (30)	13.5 (35)

^a^All strains examined were homozygous for alleles indicated and carry an integrated transgene *wIs78(ajm-1::GFP + SCMp::GFP)* to mark seam cells.

^b^Statistic significance of selected comparisons are listed in Supplementary Table 2.

^c^Number of animals (*n*) scored for each genotype is indicated in parenthesis.

^d^All strains examined were egg laying defective unless otherwise labeled with a superscript “+” denoting wild-type egg laying capability.

^e^ *hbl-1(ve18)* animals have fewer seam cells than wild type from the L2 onward, indicative of skipping L2-specific cell divisions. This strain has been previously reported to produce extra cell nuclei at the end of larval development (Abrahante et al. 2003; Abbott et al. 2005). This causes high seam cell counts for adults of this strain.

Like the single mutants, the *lin-28; hbl-1* animals showed a precocious heterochronic phenotype characterized by a reduced number of seam cells and formation of adult alae at the end of the L3 stage (Table 1, lines 1 to 6, column C). The number of seam cells in the double mutant was not much different from that of *lin-28(0)* (Table 1, column C, lines 2 and 5: *P* < 0.01). Similarly, the double mutant displayed precocious alae like that of each single mutant: patches of alae appearing at the L2 molt and full-length adult alae at the L3 molt (Supplementary Fig. 1). However, unlike single mutants, the double mutant displayed molting defects, becoming trapped in the unshed cuticle. Occasionally, when a few progeny were produced, they can be observed trapped in the deceased hermaphrodite's cuticle (Supplementary Fig. 2). As in *lin-28* mutants, vulval development in *lin-28(0); hbl-1(lf)* animals began 1 stage early with vulva precursor cells (VPC) dividing by the L2 molt. But unlike *lin-28* mutants, vulva development did not complete and appeared to arrest during morphogenesis. This explains the lack of protruding vulva that is seen in the single mutants where the vulva completes morphogenesis 1 stage early (Euling and Ambros 1996; Abrahante et al. 2003) (Supplementary Fig. 1). Arrested vulva development has been observed in animals grown on *hbl-1* RNAi (Fay et al. 1999). Also like the *lin-28* single mutant, formation of 1 or more pseudovulvae was typical, although they too arrested mid morphogenesis (Supplementary Fig. 1).

To test whether a milder version of this phenotype might result from using a weaker allele of *hbl-1*, we characterized the phenotype of a double mutant containing *hbl-1(ae23).* The *hbl-1(ae23)* allele has a small deletion at the 3′ end of the open reading frame that disrupts the 2 C-terminal dimerization zinc fingers of the protein (see Supplementary Fig. 3). The *hbl-1(ae23)* allele produces no apparent phenotype on its own (Table 1, line 4). Surprisingly, the *lin-28(ga54); hbl-1(ae23)* double mutant displayed a strong sterility phenotype, similar to that of *lin-28(ga54); hbl-1(ve18)* (Table 1, columns A and B, line 6 vs lines 2 and 3: *P* ≤ 0.0002). For all other traits, including heterochronic phenotype as well as gross body morphology, the *lin-28(ga54); hbl-1(ae23)* double mutant resembled the *lin-28(ga54); hbl-1(ve18)* double mutant.

### LIN-28 and HBL-1 are needed for gonad morphogenesis

To explore the basis of this sterility, we examined gonad development in *lin-28(0); hbl-1(lf)* double mutants. The gonad appears normal through the L2 when the first heterochronic defects of the single mutants appear (Ambros and Horvitz 1984; Abrahante et al. 2003; Lin et al. 2003). Beginning in the L3, defects in gonad development become apparent: the gonad arms do not follow their normal reflexive path to the center of the animal. By the late L4, the malformation of the uterus is observable. By adulthood, the gonads of these animals appeared disorganized with significant accumulation of yolk in the pseudocoelomic space. This pooling of yolk within the pseudocoelom indicates additional defects in the double mutant animals. It is possible that there are further abnormalities of the somatic gonad in which sheath cells surrounding the proximal gonad are unable to transport yolk from the intestine to the oocytes.

**Fig. 2.**
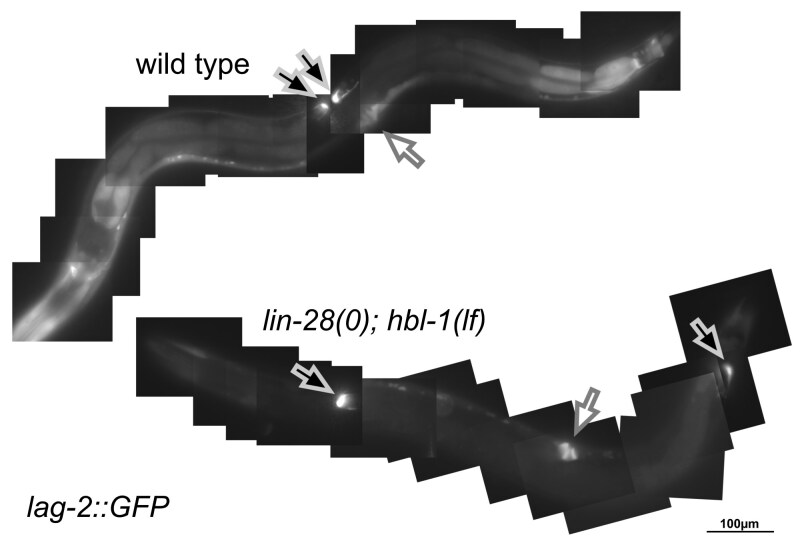
Abnormal DTC migration in *lin-28; hbl-1.* Top: wild-type *C. elegans* adult expressing *lag-2::GFP.* Black arrows, locations of fluorescing DTCs. White arrows, vulva. The DTCs are located near one another on the dorsal side opposite the vulva. Bottom: a similarly aged *lin-28; hbl-1* double mutant, also expressing *lag-2::GFP*. The DTCs are located far from each other near the head and tail.

To quantify gonad migration, we used a *lag-2::GFP* reporter which marks the DTCs as the gonad grows and migrates during the larval stages (Blelloch and Kimble 1999). Through the L2, DTC shape, position, and movement appeared normal in *lin-28(0); hbl-1(lf)* animals. Beginning in the L3, when the DTCs normally turned dorsally and reverse their direction along the anterior–posterior axis, the DTCs in *lin-28(0); hbl-1(lf)* animals migrated abnormally, often continuing toward the head or tail, and this wayward migration continued into the L4 (Fig. 2). This deviation in migration was the first visible defect identifiable in this double mutant's gonad development. Abnormal migration of the DTC of 1 arm was displayed by 24% of animals, and abnormal migration of the DTCs in both arms was displayed by 54% of animals (Table 2).

**Table 2.** Abnormal DTC migration in single and double mutants.

		A	B	C
	Genotype^a^	% normal DTC migration (*n*)^b,c,d,e^	% abnormal DTC migration of 1 arm^b,c,d,e^	% abnormal DTC migration of both arms^b,c,d,e^
1	Wild type	100 (32)	0	0
2	*lin-28(ga54)*	94 (51)	6	0
3	*hbl-1(ve18)*	91 (51)	9	0
4	*lin-28(ga54); hbl-1(ae23)*	22 (41)	24	54

^a^Strains were maintained at 20 °C.

^b^Somatic gonad defects were assessed using DIC and fluorescence microscopy.

^c^Strains examined contain *qIs56* an integrated *lag-2::GFP* reporter.

^d^Number of animals (*n*) scored for each genotype is indicated in parenthesis.

^e^Chi-square test for each comparison of line 4 vs line 2 and line 3: *P* < 0.0001.

**Fig. 3.**
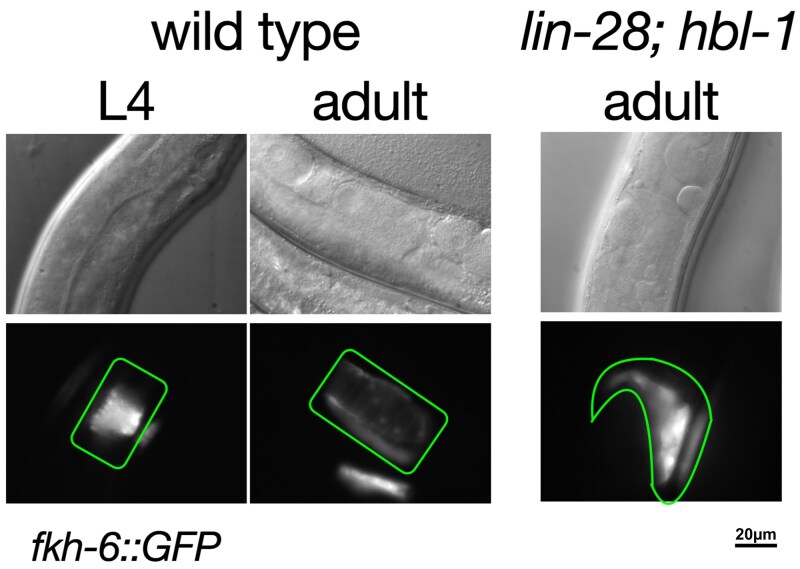
Abnormal spermatheca formation in *lin-28; hbl-1.* Left: wild-type *C. elegans* L4 and adult expressing *fkh-6::GFP* in the developing and mature spermathecae, respectively. Right: adult *lin-28; hbl-1* double mutant showing an abnormally shaped spermatheca.

The sterility phenotype observed in the double mutants could be due to defects in spermathecal development (Choi and Ambros 2019). We observed that although oocytes are formed in these double mutant animals, few gained entry into the spermatheca. The oocytes that were able to enter the spermatheca were unable to exit. To assess spermathecal development, we examined *lin-28(0); hbl-1(lf)* double mutants using a *fkh-6::GFP* reporter (Chang et al. 2004). This reporter is expressed in the spermatheca as well as spermathecal precursor cells. In wild-type animals, the reporter is consistently expressed in both spermathecae and displays the typical tube shape assumed by the spermatheca at the end of development (Fig. 3; Chang et al. 2004).

In the *lin-28(0); hbl-1(lf)* double mutant, the presentation of the reporter was markedly different from wild type in 2 ways. First, the fluorescent signal was variable, present in either both, 1, or neither arms of the gonad (Table 3). As best that could be determined, the variability in reporter expression was not due to reporter failure. The absence of reporter expression was never seen in either single mutant alone (Table 3). In the double mutant, gonad arms that displayed little to no reporter expression were also observed to be abnormal in appearance. The area of the gonad arm at which the spermatheca should be present appeared to resemble that of an earlier stage of development, in which conspicuous features of the spermatheca were not observable. When the spermatheca did not form, sometimes only a few (3 to 12) of the 24 cells that make up the spermatheca could be seen expressing the reporter. This suggests failure of spermathecal development at a stage prior to morphogenesis.

**Table 3.** *fkh-6::GFP* reporter expression in single and double mutants.

		A	B	C
	Genotype^a^	% with 2 fluorescent spermathecae (*n*)^b,c,d,e^	% with 1 fluorescent spermatheca^b,c,d,e^	% with 0 spermathecae^b,c,d,e^
1	Wild type	100 (63)	0	0
2	*lin-28(ga54)*	100 (62)	0	0
3	*hbl-1(ve18)*	100 (62)	0	0
4	*lin-28(ga54); hbl-1(ae23)*	44 (62)	38	18

^a^Strains were maintained at 20 °C.

^b^Somatic gonad defects were assessed using DIC and fluorescence microscopy.

^c^Strains examined contain *ezIs2* an integrated *fkh-6::GFP* reporter.

^d^Number of animals (*n*) scored for each genotype is indicated in parenthesis.

^e^Chi-square test for each comparison of line 4 vs line 2 and line 3: *P* < 0.0001.

The second way the reporter appeared different in the mutant is that the reporter-expressing spermatheca had an irregular morphology instead of the wild-type tube shape (Fig. 3). It sometimes exhibited what looked to be a cross-section of the spermatheca rather than the left or right side, possibly indicating improper orientation of the tissue. When the spermatheca did form correctly, sperm could be observed within it by adulthood. In most cases, the proximal gonad of an adult mutant became clogged with a mass of oocytes and oocyte fragments (Fig. 3). This observation indicated that the primary cause for the failure of fertilization of mature oocytes was defective spermathecal development. In addition to spermathecal defects, *lin-28(0); hbl-1(lf)* double mutants also displayed malformed uteri. Analysis of vulva morphology by DIC microscopy revealed that morphogenesis ceased after vulval cell invagination but before anchor cell invasion. Consequently, the formation of the uterine seam cell (utse) did not occur (Gupta et al. 2005–2018).

Because *lin-28* and *hbl-1* act during early stages of larval development, we wanted to examine the effect their loss would have on the early stages of somatic gonad development. We used a strain containing the *ckb-3p::mCherry::H2B* reporter, which is expressed in the daughters of the gonad precursor cells Z1 and Z4 in the first and second larval stage (Tenen and Greenwald 2019). We compared the number of fluorescent cells in the L1 and L2 stages of wild-type animals to *lin-28(0); hbl-1(lf)* double mutant and found no difference. While we were not able to see a cell lineage defect in these particular cells of the somatic gonad, it is possible a lineage defect occurred in other cells formed in the early larval stages or the defect simply occurred at a later developmental stage. It is also possible the defect affected the identity or behavior of certain cells. This would not affect cell number or location initially but morphological defects would result later in development.

Given that the loss of *lin-28* and *hbl-1* together dramatically impacted hermaphrodite somatic gonad development, we reasoned the male somatic gonad would also be affected. To address this question, we generated and examined males of the *lin-28(0); hbl-1(lf)* double mutant strain containing the *lag-2::GFP* reporter, which is expressed in the linker cell that leads gonad migration in males. While the loss of *lin-28* and *hbl-1* activity caused deformities in the male tail due to heterochronic defects, surprisingly, we observed no abnormal gonad migration in males and no morphological defects of the gonad whatsoever.

### LIN-28 binds directly to *lin-46*'s 5′UTR

To gain a mechanistic understanding of the relationship between *lin-28* and *hbl-1*, we further characterized the role of the protein LIN-46 in the heterochronic pathway (Pepper et al. 2004). Genetic evidence suggests that *lin-46* acts downstream of *lin-28* and upstream of *hbl-1* (Abbott et al. 2005). Mutations in *lin-28* and *lin-46* suppress each other, suggesting either that *lin-28* acts partly via *lin-46* or that they act in parallel (Pepper et al. 2004). A recent study showed that mutations in *lin-46's* 5′UTR cause a phenotype similar to *lin-28* loss-of-function, showing that the RNA-binding protein LIN-28 acts at least in part by directly regulating *lin-46* expression (Ilbay et al. 2021).

We tested whether LIN-28 can directly interact with the *lin-46* mRNA by performing the yeast 3-hybrid assay, as we had done previously to show LIN-28 binding to pre-let-7 and other pre-microRNAs (Vadla et al. 2012). We found that LIN-28 could bind the 35-nucleotide wild-type *lin-46* 5′UTR sequence, but not to a mutant 5′UTR containing a 6-nt deletion corresponding to allele *ae30* (Table 4). *lin-46(ae30)* is one of several mutations in the 5′UTR that cause a *lin-28(0)*-like phenotype (Table 1, line 9). These observations suggest that LIN-28 activity posttranscriptionally inhibits *lin-46* expression through direct binding.

**Table 4.** Yeast 3-hybrid binding of LIN-28 to *lin-46* 5′UTR.

RNA	Yeast 3-hybrid bait	Protein prey	
1		LIN-28	IRP
5′UTR	GUAAAACCAAGAAUUGUAUCAGUGGGAGUCAAUCCAAUG	+++	−
*ae30*	GUAAAACCAAGAAUU------GUGGGAGUCAAUCCAAUG	−	−
*ae31*	GUAAA---------------AGUGGGAGUCAAUCCAAUG	+/−	−
*ae40*	xXGUAAAACCAAGAAUUGU**GGG**AGUGGGAGUCAAUCCAAUG	+++	−
3′UTR	CGUGGUUGAUCUACGCUUUGCAUGA…..153nt…..AA	−	−

+++, X-gal blue after ≤ 6 h at 30°; +/−, X-gal pale blue after 24 h at 30°; −, X-gal white after 24 h at 30°.

### LIN-46 binds 2 of HBL-1's zinc fingers and inhibits HBL-1 activity in vivo

*lin-46* encodes an unusual protein whose distant relatives are involved in protein–protein interactions, although its mechanism of action in *C. elegans* is not known (Pepper et al. 2004). Ilbay and Ambros (2019) showed that *lin-46* appears to relocalize the transcription factor HBL-1 from the nucleus to the cytoplasm of seam cells. To begin to understand how the LIN-46 protein works at the molecular level, we performed a yeast 2-hybrid screen using a library of *C. elegans* cDNAs and identified HBL-1 as a directly interacting protein (Supplementary Fig. 3). We validated the interaction in vitro by GST pull down (Supplementary Fig. 3b). Although we cannot rule out the existence of other interacting partners of LIN-46, HBL-1 was the only protein we identified in the screen. The only other protein we could find that LIN-46 interacts with is itself (Supplementary Fig. 3a). Structural homology to MoeA of *E. coli* suggests that LIN-46 self-associates and likely interacts with HBL-1 as a dimer (Pepper et al. 2004).

HBL-1 is a Hunchback-like Ikaros family member with 9 zinc fingers (Fay et al. 1999). In addition to 4 zinc fingers involved in DNA binding, this family is characterized by 2 C-terminal zinc fingers involved in homodimerization and heterodimerization with other family members (McCarty et al. 2003). In yeast 2-hybrid assay experiments, LIN-46 interacted exclusively with segments of the protein containing either zinc fingers number 5 or number 9 (Supplementary Fig. 3c). Zinc finger 5 of HBL-1 is highly conserved among its relatives and is likely involved in DNA binding based on its homology to other family members. Zinc finger 9 is variable in sequence among relatives and is the most C-terminal zinc finger, 1 of the 2 ostensibly involved in dimer formation (Supplementary Fig. 3c). Interestingly, HBL-1 is unique in this family in that it does not homodimerize via its C-terminal zinc fingers (Giesecke et al. 2006). Notably, the allele *hbl-1(ae23)*, which lacks the C-terminal zinc fingers, has no discernable phenotype on its own (Table 1, line 4).

We wondered whether HBL-1 could interact with other Ikaros family members of *C. elegans* under the hypothesis that LIN-46 might disrupt a heterodimerization. We used a yeast-based interaction method that detects interactions in the cytoplasm rather than the nucleus to avoid false positives caused by the use of transcription factors as bait (Moerdyk-Schauwecker et al. 2011). We found that whereas HBL-1 could bind to LIN-46 in this assay, we detected no interaction with itself or other *C. elegans* Ikaros family members: SDZ-12, EHN-3, ZTF-16, C46E10.8, and F26F4.8 (data not shown). ZTF-16 has been shown to have a role later in the heterochronic pathway than HBL-1 (Hansen et al. 2022). We also screened a library of *C. elegans* sequences for proteins other than LIN-46 that could interact with HBL-1 and found none (data not shown).

To determine whether LIN-46 is capable of inhibiting HBL-1 activity in vivo, we took advantage of a situation in which *hbl-1* is inappropriately expressed. In some *C. elegans* motor neurons, *hbl-1* is transcriptionally repressed by the transcription factor UNC-55 (Thompson-Peer et al. 2012). When *unc-55* is mutant, HBL-1 is ectopically expressed, which leads to inappropriate rewiring of the synapses of these motor neurons, resulting in the characteristic “uncoordinated” phenotype of *unc-55* mutants. If LIN-46 is an inhibitor of HBL-1 activity in vivo, ectopic expression of LIN-46 in neurons of *unc-55* mutants should suppress the ectopic rewiring and alleviate the uncoordinated phenotype.

We previously reported that a *lin-46::GFP* translational fusion is visible only periodically in the lateral and ventral epidermis, with no discernible fluorescence in the surrounding hypodermal syncytium or other sites (Pepper et al. 2004). We were unsuccessful when attempting to express this fusion protein constitutively or more broadly in hypodermal cells using heterologous promoters (e.g. *col-10*), suggesting that LIN-46 accumulation is restricted temporally and spatially by a post-translational mechanism (Supplementary Fig. 4a). However, we were able to express LIN-46:GFP constitutively in cells of the nervous system using the *rgef-1* promoter (Supplementary Fig. 4b) (Chen et al. 2011).

*unc-55* mutants have a characteristic coiling behavior when moving backward, and loss-of-function mutations in *hbl-1* partially suppress this phenotype (Thompson-Peer et al. 2012). We found that ectopic expression of LIN-46 in neurons suppressed the *unc-55* mutant's coiling to the same degree as a *hbl-1* mutation, whereas expressing a paralog of LIN-46 (MOC-1) did not have this effect (Supplementary Fig. 5a). Coiling is due to a defect in neuromuscular synapse formation, which can be visualized using reporters. *unc-55* mutants have fewer ventral synapses whereas an *unc-55; hbl-1* double mutant has significantly more ventral synapses (Thompson-Peer et al. 2012). By counting the number of synapses, we determined that ectopic LIN-46 also raises the number of ventral synapses in a *unc-55* mutant to the same degree as loss of *hbl-1* (Supplementary Fig. 5b).

Finally, to test whether zinc fingers of HBL-1 could bind to and inhibit LIN-46 in vivo, we expressed a C-terminal domain of HBL-1 with LIN-46-binding activity (labeled with an asterisk in Supplementary Fig. 3), in developing larvae using the *col-10* promoter. We found that ectopically expressed HBL-1 C-terminal domain caused a *lin-46(0)*-like phenotype, producing gaps in adult later alae (Supplementary Table 1). The notable difference was that although *lin-46(0)* is cold-sensitive, expression of the HBL-1 C-terminal domain was not (Supplementary Table 1). Our results therefore suggest that LIN-46 directly inhibits HBL-1 activity by binding 2 of its zinc fingers, 5 and 9, possibly interfering with DNA binding.

### *lin-28* acts only in part through *lin-46* to have its effect on gonad development

Because the sterility of the *lin-28(0); hbl-1(lf)* double mutant was unlike either mutant alone, it is possible that loss of *lin-28* further reduced the activity of a *hbl-1* hypomorphic allele. Previous work from the laboratory suggested that *lin-28* might act via *hbl-1*'s 3′UTR (Vadla et al. 2012). However, because the results presented above suggest that *lin-28* regulates *hbl-1* via *lin-46*, we characterized *lin-46*'s role with respect to the *lin-28; hbl-1* gonad phenotype by genetic analysis. Specifically, we sought to determine whether *lin-28* acts entirely or in part through *lin-46* to regulate *hbl-1*.

Mutations in *lin-28* and *lin-46* cause opposite heterochronic phenotypes and fully suppress each other's timing defect when combined (Pepper et al. 2004). There is a significant difference in the penetrance and expressivity of their phenotypes, indicating that the genes do not act in a simple linear pathway (Pepper et al. 2004). Additionally, alleles of *lin-46* are not able to suppress *hbl-1* hypomorphic allele phenotypes, which resemble those of *lin-28* null mutants (Pepper et al. 2004; Abbott et al. 2005). To test whether *lin-46* null would suppress the sterility phenotype of the *lin-28(0); hbl-1(lf)* double mutant, we generated a *lin-28(0); lin-46(0); hbl-1(lf)* strain. We found the sterility phenotype was partially rescued in this strain (Table 1, column A, line 7 vs line 5: *P* < 0.0001). Suppression occurred with both weak and strong *hbl-1* alleles, although fertility was not fully restored for either (Table 1, column A, lines 8 vs line 6: *P* < 0.0001). The brood sizes of fertile triple mutants were similar to those seen in the *lin-28*; *hbl-1* double mutants (Table 1, column B, lines 3 to 8).

The *lin-46(ae30)* allele, which has a 6-bp deletion in its 5′UTR that prevented LIN-28 binding (Table 4), produced a precocious phenotype similar to a *lin-28* null allele (Table 1, line 9). Ilbay and Ambros (2019) identified that the lesion of this allele is similar in sequence to 5′UTR deletions of *lin-46* (Ilbay et al. 2021). If *lin-28* supports *hbl-1* expression or activity via *lin-46,* then *lin-46(ae30)* when combined with a *hbl-1* hypomorphic allele should cause sterility. Remarkably, the fertility of *lin-46(ae30); hbl-1(ve18)* was no lower than that of *hbl-1(ve18)* alone (Table 1, column A, line 10 vs line 3: *P* = 0.13), and significantly higher than that of the *lin-28(0); hbl-1(ve18)* animals (Table 1, column A, line 10 vs line 5: *P* < 0.0001). Furthermore, we saw 100% fertility when *lin-46(ae30)* was combined with the weaker *hbl-1(ae23)* allele (Table 1, compare lines 6 and 11), which is significantly different from the sterility we observed with the *lin-28(0); hbl-1(ae23)* double mutant (Table 1, column A, line 11 vs line 6: *P* < 0.0001). This suggests that a significant fraction of *lin-28*'s effect on *hbl-1* occurs independently of *lin-46*.

### HBL-1 activity is necessary for normal gonad development

Because a null allele of *hbl-1* is embryonic lethal, it was difficult to directly address whether *lin-28* and *hbl-1* act in a linear or a branched pathway to affect gonad development. To resolve this issue, we analyzed gonad development after supplying HBL-1 activity during embryogenesis and removing it during larval development. To control when HBL-1 is active, we utilized the AID system where the addition of the plant hormone auxin to NGM causes in vivo degradation of proteins containing the auxin-binding degron (Zhang et al. 2015; Hills-Muckey et al. 2021).

We generated a strain that contained the endogenous *hbl-1* locus tagged at its C-terminus with the degron sequence (*hbl-1::AID*). Without auxin, these animals showed a wild-type phenotype, whereas, when grown on auxin-containing media, they displayed a phenotype that had reduced fecundity and fewer seam cells like that of the *hbl-1(ve18)* allele where HBL-1 activity is reduced (Table 1, lines 12 and 13). To maximally reduce *hbl-1* activity during larval development, we grew newly hatched *hbl-1::AID* animals on media containing auxin and *E. coli* expressing *hbl-1* dsRNA (*hbl-1* RNAi). Eggs from this strain were transferred onto auxin + RNAi plates at the 3-fold stage of development and grown to adulthood. Under these conditions, we observed that 50% of the *hbl-1::AID* animals displayed a sterility phenotype with fertile animals producing a reduced number of offspring (Table 1, line 15). Many sterile animals of adult age were found to display abnormal gonad morphology, like that of the *lin-28(0); hbl-1(lf)* double mutants, with no fertilized embryos present. Thus, reduction of *hbl-1* activity alone during larval development is sufficient to affect gonad morphogenesis. Removal of *lin-28* activity from *hbl-1::AID* animals reared on *hbl-1* RNAi and auxin increased the severity of the sterility defect, with none of the animals being fertile (Table 1, line 16). These observations support a model whereby *lin-28* regulates the expression and/or activity of *hbl-1*, and *hbl-1* is the proximal gene responsible for normal gonad development.

As demonstrated above, *lin-46* is able to suppress the sterility phenotype of the *lin-28(0); hbl-1(lf)* double mutants. We sought to determine whether *lin-46* would still be able to suppress the sterility phenotype of the *lin-28(0); hbl-1::AID* double mutant. The removal of *lin-46* from the *lin-28(0); hbl-1::AID* resulted in partial rescue of the sterility phenotype. Fertility was restored to 27% in *lin-28(0); lin-46(0); hbl-1::AID* triple mutants (Table 1, column A, line 17 vs 16: *P* = 0.0007). These results support the idea that *hbl-1* is the most proximal acting factor in the heterochronic pathway for the control of somatic gonad development and that *lin-28* acts in part through *lin-46* to exert positive regulation of *hbl-1*.

## Discussion

Here, we make use of an unexpected gonad morphogenesis phenotype to shed light on so far unresolved molecular relationships among heterochronic gene regulators. Although gonad defects have been described in some heterochronic mutants, the extent of gonad deformity and sterility seen here is unprecedented in this pathway, providing an opportunity to investigate certain regulatory relationships among these genes. Although the gonad phenotype is not connected in an obvious way with developmental timing, our findings, along with new evidence of direct interactions, shed light on mechanistic relationships.

We found that when combining loss-of-function alleles of *lin-28* and *hbl-1*, which on their own cause heterochronic phenotypes in the hypodermis, there occurred an unexpected level of sterility due to abnormal gonad growth and development. In addition to abnormal DTC migration seen in some other developmental timing mutants, developmental failure of the hermaphrodite spermatheca is severe (Fig. 1). This phenotype is not merely additive: although single mutants of *lin-28* and *hbl-1* occasionally had 1 arm of the gonad display abnormal migration, in more than half of the double mutants both arms migrated abnormally, and it was uncommon to see an animal with normal gonad migration (Table 2). Furthermore, neither single mutant ever lacked a spermatheca entirely, but again more than half of the double mutant animals completely lacked 1 or both structures (Table 3).

The heterochronic genes are characterized by temporal cell fate transformations, substituting 1 stage-specific fate for another. It is difficult to determine whether cell fate transformations like this occurred in the gonad of *lin-28; hbl-1* double mutants: gonad development appeared normal through the L2 when most of the lineages producing gonadal cells are complete. We have not been able to identify the initial cellular defect that leads to the catastrophic failure of gonad morphogenesis in these mutants. The first defect we observed was the aberrant migration of DTCs at the start of the L3. Although *lin-28* and *hbl-1* have not been previously implicated in governing DTC migration, later-acting heterochronic genes that act in parallel with or downstream of both in the heterochronic pathway have been shown to have a significant role in DTC migration in late larval development (Huang et al. 2014; Cecchetelli and Cram 2017). It is believed that these later-acting genes control signaling within the DTCs themselves, so learning where *lin-28* and *hbl-1* are required to have an effect on normal gonad morphogenesis—whether in the DTCs, the hypodermis, or other sites—will be important to understand this phenomenon.

It is far less clear how gonad morphogenesis, specifically spermathecal and uterine development, are governed by heterochronic gene activity since single gene mutants with strong hypodermal phenotypes show no such defects. Whether these genes act within the hypodermis to affect gonad morphogenesis or within the gonad itself must be determined. Still, any theory explaining how the early-acting heterochronic genes impact gonad development of the hermaphrodite must take into account that male gonad development appears normal, despite their high-penetrance impacts on hypodermal development in males.

We initiated these studies to clarify the relative roles of *lin-28*, *lin-46*, and *hbl-1* in the heterochronic pathway. Previous genetic studies indicate that the activities of multiple factors of the heterochronic pathway converge onto *hbl-1*, which appears to be the most proximal acting gene involved in controlling cell fates of the L2 (Abbott et al. 2005; Ilbay and Ambros 2019). Indeed, we provide new evidence for direct interactions between the RNA-binding protein LIN-28 and the 5′UTR of *lin-46* (Table 4), and between LIN-46 and HBL-1 zinc fingers (Supplementary Fig. 3), also confirming that LIN-46 can inhibit HBL-1 activity in vivo (Supplementary Fig. 5). Along with prior genetic evidence, these data are consistent with a model whereby *lin-28* acts via negative regulation of *lin-46* to positively regulate *hbl-1* in a linear pathway (Supplementary Fig. 6). However, other lines of evidence indicate that this model does not account for a *lin-46-*independent regulation of *hbl-1* by *lin-28* (Pepper et al. 2004; Vadla et al. 2012). Here, we find further indications that *lin-28* does not act solely through *lin-46* to regulate *hbl-1*. Particularly, a mutation in the 5′UTR of *lin-46* that abrogates LIN-28 binding does not phenocopy the *lin-28(0); hbl-1(lf)* sterility in that most of the *lin-46(Δutr); hbl-1(lf)* animals were fertile with normal gonads (Table 1, compare lines 5 and 10 and lines 6 and 11). We conclude, therefore, that *lin-28* acts in a branched fashion to influence *hbl-1* activity, possibly posttranscriptionally (via its 3′UTR) and posttranslationally (via LIN-46) (Supplementary Fig. 6). Further studies could assess the relative weights of these 2 branches.

Our results show that *hbl-1* is required during larval development for normal gonad morphogenesis. The work of Ilbay and Ambros (2019) and our own unpublished findings show that deletion of microRNA regulatory sites in the 3′UTR of *hbl-1* does not impact gonad development. This difference suggests that the mere presence of HBL-1 and not its temporal regulation matters for normal fertility. The threshold for the level of HBL-1 activity required for normal somatic gonad development appears to be lower than the level of activity required for correct developmental timing in the hypodermis as hypomorphic mutants produce a heterochronic effect while gonad development is normal.

*hbl-1*, which encodes an Ikaros family transcription factor, is known to be pleiotropic in *C. elegans* development. In addition to its larval developmental timing function, *hbl-1* has been shown to have roles in late embryonic development, synaptic remodeling, and dauer formation (Fay et al. 1999; Karp and Ambros 2011; Thompson-Peer et al. 2012). Where these roles differ from *hbl-1*'s effect on the gonad is that we see a role for other heterochronic genes in the later phenotypes. In other words, we believe that normal gonad development does not merely require *hbl-1* and no other heterochronic pathway members like, e.g., late embryonic hypodermal development. Rather, gonad development requires multiple members of at least 1 branch of the heterochronic pathway, suggesting a more extensive role for the developmental timing genes in gonad development than previously known.

Finally, we recently observed that *Caenorhabditis briggsae* orthologs of *C. elegans* heterochronic genes show mutant phenotypes that differ in significant ways from those of *C. elegans* (Ivanova and Moss 2023). Specifically, several of these *C. briggsae* mutants show a gonad disintegration phenotype that closely resembles the gonad phenotype we describe here. The *C. briggsae* genes with apparent roles in gonad development include *Cbr-lin-28*, *Cbr-lin-46*, *Cbr-hbl-1*, and *Cbr-lin-41*. Compared with their *C. elegans* counterparts, these genes show much weaker heterochronic phenotypes in the hypodermis, whereas their roles in gonad development are more apparent, suggesting significant evolutionary drift in their activities since the last common ancestor of *C. elegans and C. briggsae*. Nevertheless, despite this drift, we note that the heterochronic genes of both species have clear roles in both developmental timing and gonad morphogenesis, suggesting an enduring association between these aspects of development.

## Supplementary Material

jkaf170_Supplementary_Data

## Data Availability

Strains and plasmids are available upon request. The authors affirm that all data necessary for confirming the conclusions of the article are present within the article, figures, and tables. Supplemental material available at G3 online.
