# Supplementary material for: Somatic gonad morphogenesis in C. elegans requires the heterochronic pathway acting through HBL-1
Source: G3 (Bethesda). 2025 Jul 28;15(10):jkaf170. doi: 10.1093/g3journal/jkaf170 (PMC12506653; doi:10.1093/g3journal/jkaf170)
Supplement: jkaf170_Supplementary_Data [file jkaf170_supplementary_data.docx]

***Supplementary Information***

**Supplementary Table 1. *lin-46(0)-*like phenotype caused by ectopic HBL-1 C-terminal fragment*.***

|  | Genotype and growth temperature | % with gaps in adult alae (*n*)^a,b^ |
| --- | --- | --- |
|  | wild type |  |
| 1 | 15° | 0 (20) |
| 2 | 20° | 0 (20) |
| 3 | 25° | 0 (22) |
|  | *lin-46(0)* |  |
| 4 | 15° | 100 (20) |
| 5 | 20° | 29 (24) |
| 6 | 25° | 4 (24) |
|  | *col-10p::hbl-1C-term.* |  |
| 7 | 15° | 5 (21) |
| 8 | 20° | 45 (20) |
| 9 | 25° | 23 (22) |

^a^ Alae gaps observed in young adult animals covered one to several seam cells.
^b^ Number of animals (n) scored for each genotype is indicated in parenthesis; one side scored per animal.

**Supplementary Table 2. Table 1 statistics.**

| **line #s** | **genotypes compared** | **% fertile**^a ,b^ | **avg. brood size**^a,b^ | **number of seam cells**^a,b^ |
| --- | --- | --- | --- | --- |
| 5  2 | *lin-28(ga54); hbl-1(ve18)*  *lin-28(ga54)* | <0.0001 | <0.0001 | **0.0082** |
| 5  3 | *lin-28(ga54); hbl-1(ve18)*  *hbl-1(ve18)* | <0.0001 | <0.0001 | n/a |
| 6  2 | *lin-28(ga54); hbl-1(ae23)*  *lin-28(ga54)* | <0.0001 | <0.0001 | **0.2647** |
| 6  3 | *lin-28(ga54); hbl-1(ae23)*  *hbl-1(ve18)* | <0.0001 | 0.0002 | n/a |
| 5  6 | *lin-28(ga54); hbl-1(ve18)*  *lin-28(ga54); hbl-1(ae23)* | **0.0882** | n/a | n/a |
| 7  5 | *lin-28(ga54); lin-46(ma174); hbl-1(ve18)*  *lin-28(ga54); hbl-1(ve18)* | <0.0001 | **0.8289** | <0.0001 |
| 8  6 | *lin-28(ga54); lin-46(ma174); hbl-1(ae23)*  *lin-28(ga54); hbl-1(ae23)* | <0.0001 | <0.0001 | <0.0001 |
| 10  3 | *lin-46(ae30); hbl-1(ve18)*  *hbl-1(ve18)* | **0.1293** | <0.0001 | <0.0001 |
| 10  5 | *lin-46(ae30); hbl-1(ve18)*  *lin-28(ga54); hbl-1(ve18)* | <0.0001 | **0.2949** | <0.0001 |
| 6  11 | *lin-28(ga54); hbl-1(ae23)*  *lin-46(ae30); hbl-1(ae23)* | <0.0001 | <0.0001 | 0.0005 |
| 13  12 | *hbl-1(AID)*  *hbl-1(AID)*, no auxin | **>0.9999** | <0.0001 | <0.0001 |
| 13  3 | *hbl-1(AID)*  *hbl-1(ve18)* | n/a | **0.9707** | <0.0001 |
| 15  3 | *hbl-1(AID+RNAi)*  *hbl-1(ve18)* | <0.0001 | <0.0001 | n/a |
| 15  5 | *hbl-1(AID+RNAi)*  *lin-28(ga54); hbl-1(ve18)* | <0.0001 | **0.2376** | <0.0001 |
| 16  15 | *lin-28(ga54); hbl-1(AID+RNAi)*  *hbl-1(AID+RNAi)* | <0.0001 | n/a | 0.0011 |
| 16  5 | *lin-28(ga54); hbl-1(AID+RNAi)*  *lin-28(ga54); hbl-1(ve18)* | **0.1547** | <0.0001 | 0.0005 |
| 17  16 | *lin-28(ga54); lin-46(ma174); hbl-1(AID+RNAi)*  *lin-28(ga54); hbl-1(AID+RNAi)* | 0.0007 | <0.0001 | <0.0001 |
| 17  15 | *lin-28(ga54); lin-46(ma174); hbl-1(AID+RNAi)*  *hbl-1(AID+RNAi)* | **0.0769** | **0.0506** | <0.0001 |
| 17  7 | *lin-28(ga54); lin-46(ma174); hbl-1(AID+RNAi)*  *lin-28(ga54); lin-46(ma174); hbl-1(ve18)* | 0.0019 | **0.3015** | **0.0052** |

^a^ Bold type: not significant (Bonferroni correction applied for each outcome variable: *p*<0.05/19=0.0026).

^b^ Statistical tests: % fertile, Fisher’s Exact test; avg. brood size, unpaired parametric *t*-test; number of seam cells, nonparametric Mann-Whitney.

**
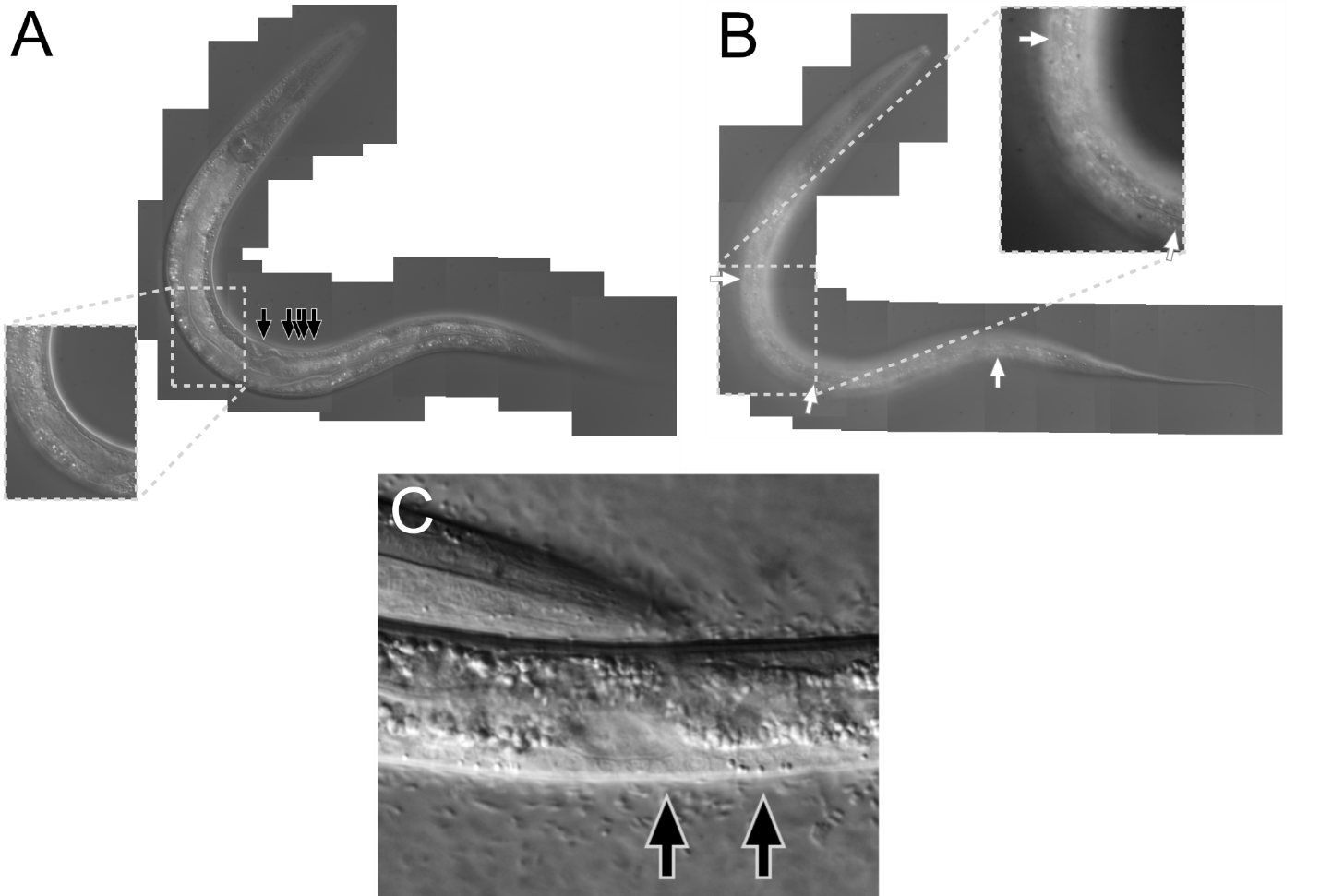
**

**Supplementary Figure 1. Phenotypes displayed by *lin-28; hbl-1* larvae. A.** *lin-28; hbl-1 L3* larvae displaying advanced vulva morphogenesis and abnormal multivulva (Muv) morphology (black arrows). **Inset Box** showing stage in larval development based on gonad arm position. **B.** *lin-28; hbl-1* L3 larvae displaying full adult-specific lateral alae (white arrows). **Inset Box** showing magnified view of alae. **C.** Individual *lin-28; hbl-1* larvae at L2 molt displaying precocious VPC divisions (black arrows).

**
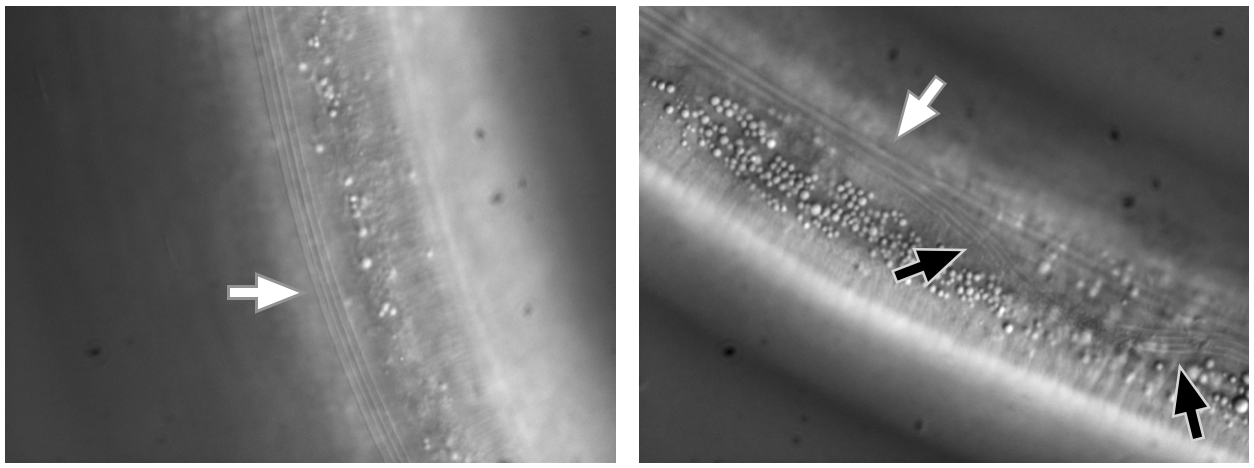
**

**Supplementary Figure 2. Molting defects displayed by *lin-28; hbl-1* mutant. A.** Wild type *C. elegans* adult displaying alae on single cuticle (white arrow). **B.** A similarly-aged *lin-28; hbl-1* double mutant displaying alae ridges from older, unshed cuticle (white arrow) as well as a newer cuticle that can be seen underneath (black arrows).


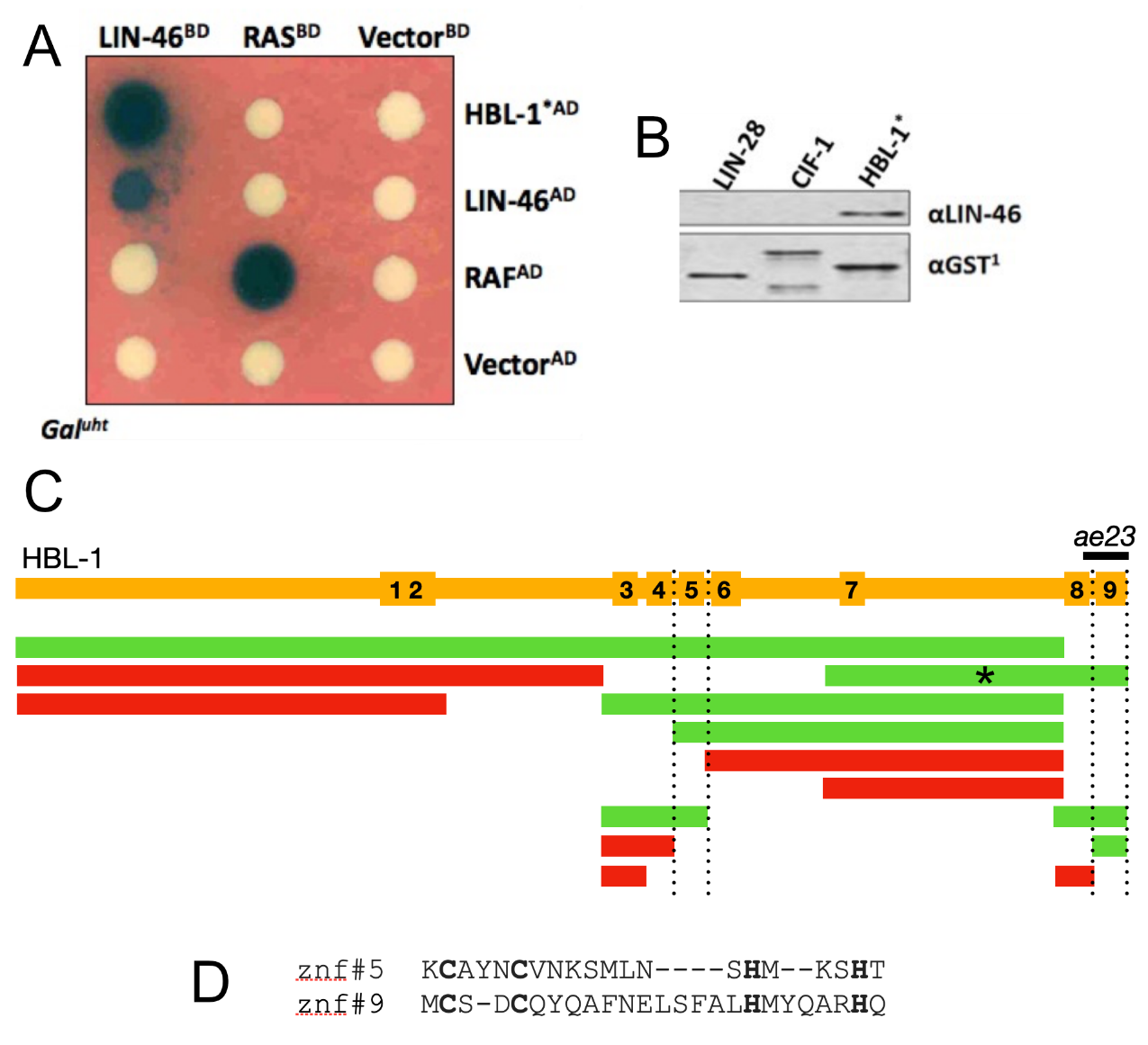
 **Supplementary Figure 3. Interaction of HBL-1 with LIN-46. A,** Yeast two-hybrid tests of binding among LIN-46, HBL-1, and control proteins (RAS and RAF). HBL-1* is a C-terminal fragment of HBL-1 indicated by the asterisk in panel C. Dark blue colonies are indicative of an interaction, white means no interaction. BD, DNA binding domain. AD, activation domain. **B,** Immunoblots of a GST pull-down experiment where proteins labeled across the top were fused with GST and mixed with unfused LIN-46. **Bottom panel:** All three GST fusions could be detected in the pull-down by anti-GST antisera. **Top panel:** Only the HBL-1* fragment pulled down LIN-46.
**C,** Summary of yeast two-hybrid interactions between LIN-46 and portions of HBL-1. A schematic of the HBL-1 protein sequence showing the locations of 9 zinc fingers and indicating the sequences missing in allele *ae23* (black bar). Green: HBL-1 portions that show a strong positive interaction with LIN-46 in a yeast two-hybrid assay. Red: Portions that show no interaction with LIN-46. **D,** The sequences of zinc fingers 5 and 9 aligned by their cysteine and histidine residues showing their dissimilarity.

**
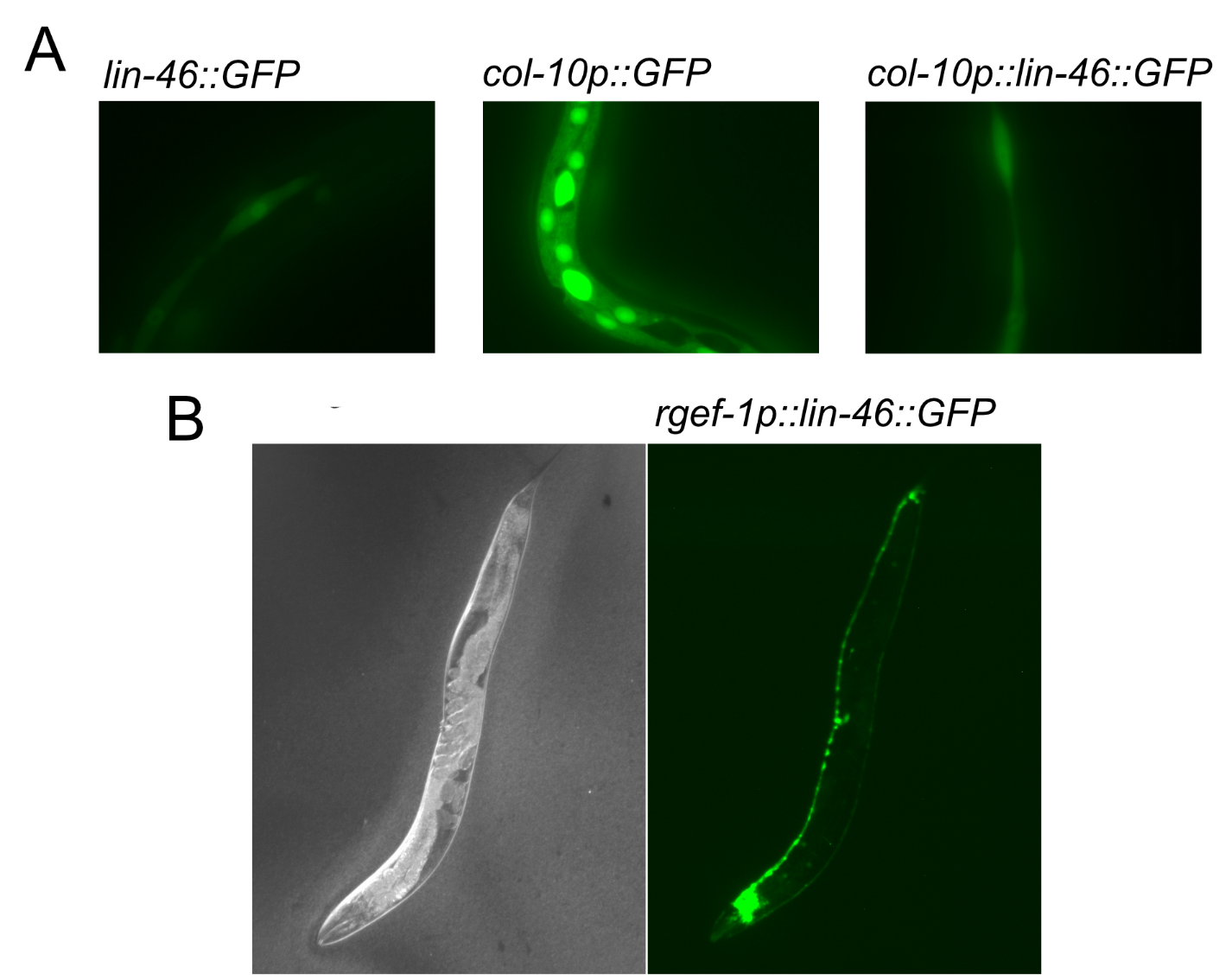
**

**Supplementary Figure 4. Ectopic expression of LIN-46. A,** Fluorescence micrographs of late-stage larvae expressing the indicated transgenes. For both *lin-46::GFP* and *col-10p::lin-46::GFP*, fluorescence is seen dimly in seam cells at the time of the molts, whereas *col-10p::GFP* is bright continuously throughout the hypodermis. **B,** A DIC image (**left**) and a fluorescence image (**right**) of an adult expressing *rgef-1::lin-46::GFP*. Expression is continuous throughout the nervous system.


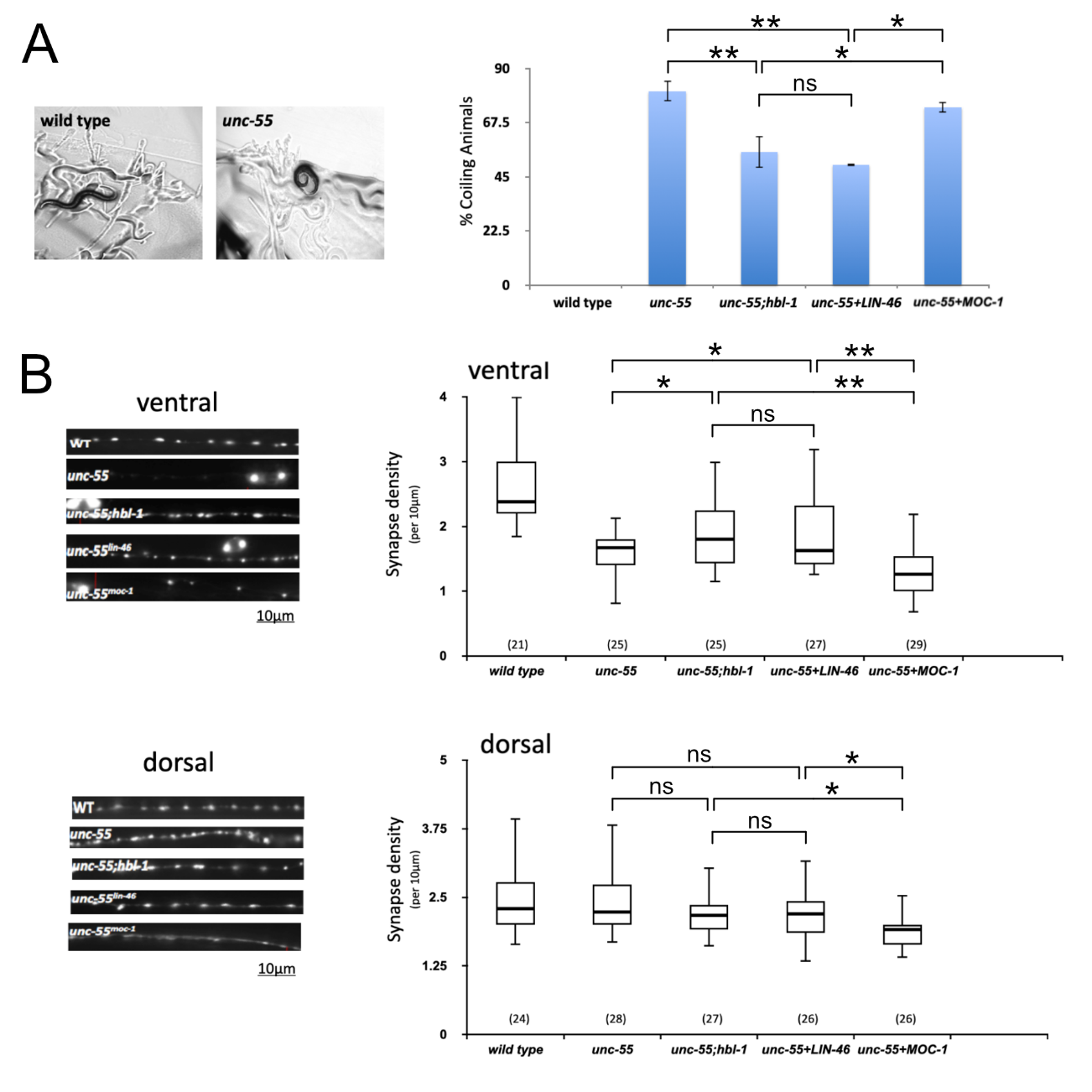


**Supplementary Figure 5. LIN-46 inhibits HBL-1 activity in vivo. A, Left**, A comparison of the movement of wildtype and *unc-55* mutant animals. Wild type backs up in a sinusoidal motion, whereas *unc-55* mutants move backward with difficulty and frequently coil. A *hbl-1* mutation decreases the coiling of *unc-55* mutants (Thompson-Peer et al. 2012). **Right**, Percentages of animals of different genotypes that display coiling behavior. LIN-46 and MOC-1 (a LIN-46 paralog) were ectopically expressed in *unc-55* mutants. *, *p*<0.01 by Fisher's exact test. ns, not significant. **B,** *unc-55* mutants have fewer ventral synapses but the normal number of dorsal synapses. A *hbl-1* mutation increases the number of ventral synapses of *unc-55* mutants (Thompson-Peer et al. 2012). Synapses are visualized using the endophilin marker *unc-57:GFP*. **Left**, representative images of ventral and dorsal synapses in strains of different genotypes. **Right**, quantitation of synapse density per 10 microns of strains of different genotypes. **, *p*<0.01, *, *p*<0.05 by *t-*test. ns, non-significant.


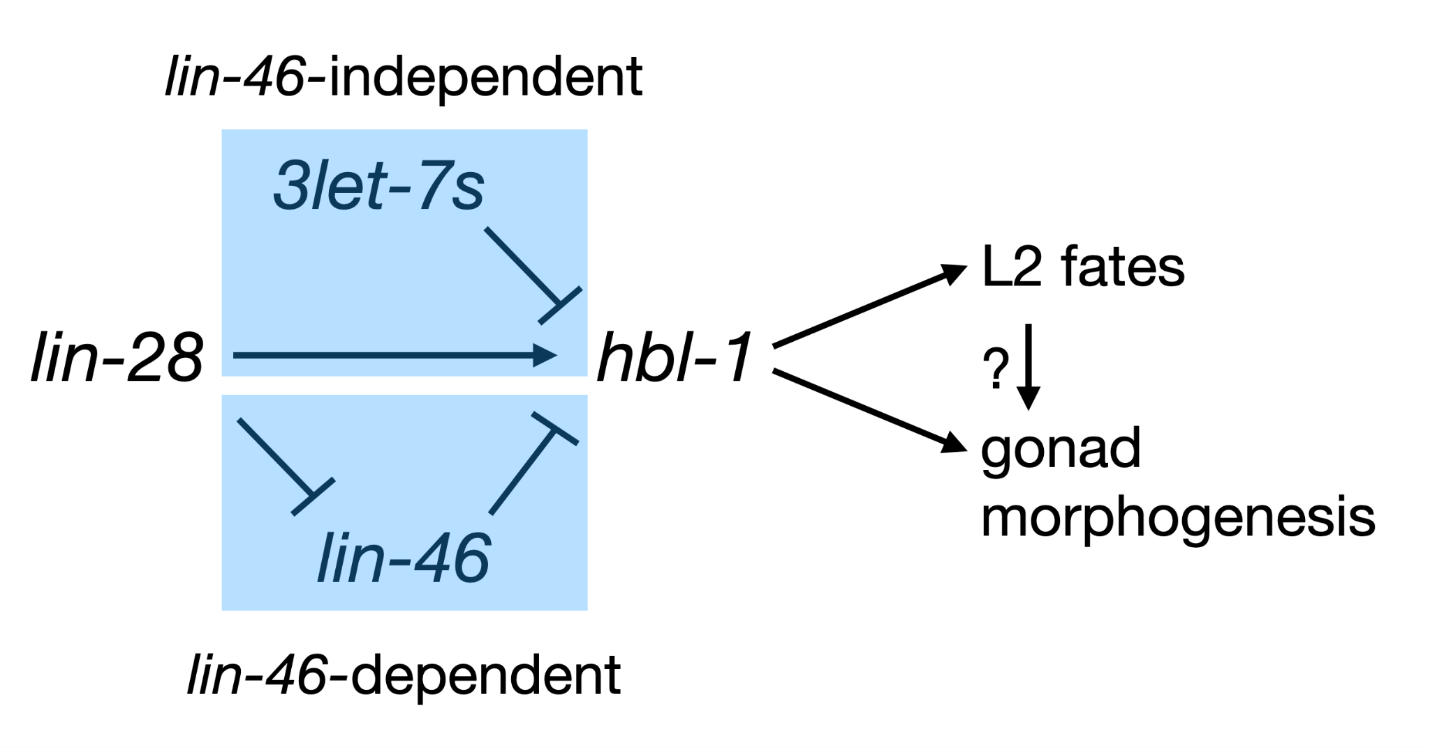


**Supplementary Figure 6. A model for the regulation of *hbl-1* in the heterochronic pathway.** We propose a branched regulation of *hbl-1* by *lin-28*: A *lin-46-*dependent branch whereby LIN-46 directly inhibits the HBL-1 protein by binding its zinc fingers, and LIN-28 inhibits synthesis of LIN-46 by binding its 5’ UTR; and a *lin-46-*independent branch. *hbl-1* is the most proximal regulator to L2 cell fates and gonad morphogenesis. Whether gonad morphogenesis is directly regulated by *hbl-1* or a consequence of its control of L2 cell fates is not known.
